# Effects of Long-Term Cryopreservation on the Transcriptomes of Giant Grouper Sperm

**DOI:** 10.3390/genes15040523

**Published:** 2024-04-22

**Authors:** Xiaoyu Ding, Yongsheng Tian, Yishu Qiu, Pengfei Duan, Xinyi Wang, Zhentong Li, Linlin Li, Yang Liu, Linna Wang

**Affiliations:** 1 State Key Laboratory of Mariculture Biobreeding and Sustainable Goods, Yellow Sea Fisheries Research Institute, Chinese Academy of Fishery Sciences, Qingdao 266071, China; dingxy0503@163.com (X.D.);; 2 Laboratory for Marine Fisheries Science and Food Production Processes, Qingdao Marine Science and Technology Center, Qingdao 266237, China; 3 Hainan Innovation Research Institute, Chinese Academy of Fishery Sciences, Sanya 572000, China

**Keywords:** *Epinephelus lanceolatus*, sperm freezing damage, transcriptome analysis

## Abstract

The giant grouper fish (*Epinephelus lanceolatus*), one of the largest and rarest groupers, is a fast-growing economic fish. Grouper sperm is often used for cross-breeding with other fish and therefore sperm cryopreservation is important. However, freezing damage cannot be avoided. Herein, we performed a transcriptome analysis to compare fresh and frozen sperm of the giant grouper with frozen storage times of 0, 23, 49, and 61 months. In total, 1911 differentially expressed genes (DEGs), including 91 in El-0-vs-El-23 (40 upregulated and 51 downregulated), 251 in El-0-vs-El-49 (152 upregulated and 69 downregulated), and 1569 in El-0-vs-El-61 (984 upregulated and 585 downregulated), were obtained in the giant grouper sperm. DEGs were significantly increased at 61 months of cryopreservation (*p* < 0.05). GO and KEGG enrichment analyses of the DEGs revealed significant enrichment in the pilus assembly, metabolic process, MAPK signaling pathway, apoptosis, and P53 signaling pathway. Time-series expression profiling of the DEGs showed that consistently upregulated modules were also significantly enriched in signaling pathways associated with apoptosis. Four genes, *scarb1*, *odf3*, *exoc8*, and *atp5f1d*, were associated with mitochondria and flagella in a weighted correlation network analysis. These genes may play an important role in the response to sperm freezing. The experimental results show that long-term cryopreservation results in freezing damage to the giant grouper sperm. This study provides rich data for studies of the mechanism underlying frozen fish sperm damage as well as a technical reference and evaluation index for the long-term cryopreservation of fish sperm.

## 1. Introduction

The giant grouper fish (*Epinephelus lanceolatus*) is the largest species in the grouper family. It is also an important and rare mariculture fish in China. However, male and female grouper development is not synchronized and the number of males is small. Thus, sperm cryopreservation is very important. In aquaculture, sperm cryopreservation not only preserves good germplasm but is also conducive to fish breeding and provides year-round sperm to expand the breeding cycle. Sperm freezing using cryopreservation technology has been established for several grouper species, including the giant grouper (*E. lanceolatus*) [1], potato grouper (*E. tukula*) [2], and seven-band grouper (*E. septemfasciatus*) [3].

Under ultra-low-temperature conditions, the protein, enzymes, metabolism, and biochemical functions of frozen cells themselves are almost completely stagnant, and the organism is in a stationary state [4]. If the body structure of the organism remains intact, it will be restored after rewarming. However, cryoinjury is almost impossible to avoid. Sperm morphology and motility speed are correlated with the sperm fertilization rate [5]. Ultra-low temperatures can also cause oxidative stress, resulting in apoptosis and mitochondrial damage, thereby directly affecting sperm quality [6].

There is evidence that long-term cryopreservation reduces sperm motility, plasma membrane integrity, and sperm enzyme activity and increases seminal plasma enzyme activity, which will have a certain impact on the ultrastructure of giant grouper sperm; furthermore, these effects increase gradually with the extension of the freezing time [7]. These findings are consistent with those of other studies on sperm freezing damage in the giant grouper species [8]. However, the underlying molecular mechanisms remain unclear. High-quality sperm is important for actual production. There is also extensive evidence that cryopreserved freezing not only affects the sperm structure but also affects some mRNAs, thus damaging related protein function [9]. In an analysis of mRNA and lncRNA transcriptome sequencing in cryoinjuries in frozen–thawed panda (*Ailuropoda melanoleuca*) sperm, one study found that most of the differential genes were correlated with sperm membrane function, metabolism, and apoptosis, which also verified the various damage of sperm induced by cryogenic freezing [10]. DE mRNAs and miRNAs are heavily involved in boar sperm’s response to environment stimuli, apoptosis, and metabolic activities. The differences in expression also reflect the various structural and functional changes in sperm during cryopreservation [11]. Therefore, transcriptome analyses of differentially expressed genes (DEGs) between fresh sperm and sperm subjected to long-term cryopreservation are important to gain a more comprehensive and systematic understanding of the molecular mechanisms underlying the effects of ultra-cold freezing.

The contribution of mRNAs to the regulation of the cold response in cryopreserved giant grouper sperm has yet to be elucidated. Here, we employed a high-throughput sequencing approach to explore the mRNA expression profiles of fresh and frozen–thawed giant grouper sperm to investigate the alterations in global gene expression in response to cryopreservation, with the goal being to better understand the molecular mechanisms behind fish sperm cryoinjury.

## 2. Materials and Methods

### 2.1. Ethics Statement

This study was approved by the Animal Care and Use Committee of the Yellow Sea Fisheries Research Institute (Qingdao, China).

### 2.2. Sperm Collection

Giant grouper sperm samples were collected from Laizhou Ming Bo Aquatic Co., Ltd. (Laizhou city, China) from a healthy giant grouper male. This giant grouper was cultivated in indoor recirculation systems at 25–30 °C (body length, 1.4 m; body weight, 58 kg). During the giant grouper breeding season from July to September, fresh semen was collected in August 2022, and sperm were cryopreserved in September 2020, July 2018, and July 2017. Frozen storage times were 0, 23, 49, and 61 months. After administering artificial oxytocin, the fish body was wrapped in a clean towel and gently squeezed, and fresh semen was collected into a 50 mL centrifuge tube by squeezing. The semen needed to be white or milky white to ensure that there was no feces or urine in the semen. Samples were refrigerated in a 4 °C refrigerator after collection. The sperm samples with a motility greater than or equal to 85% were selected for the following cryopreservation test.

### 2.3. Sperm Cryopreservation and Thawing

The cryopreservation protocol used in the sperm freezing and thawing process was obtained from our laboratory [1]. Frozen semen samples from three time periods were randomly selected, and three frozen sperm tubes were selected from each time period for thawing. Meanwhile, fresh semen from mature males was collected as a control group sample. Semen supplemented with the sperm dilution ELS3 and 15% DMSO + 10% FBS antifreeze was divided in 2 mL cryovials and equilibrated for 5 min at room temperature (20–25 °C). The cryovials were placed into cloth bags, suspended in liquid nitrogen at a height of 10 cm for 10 min, equilibrated for 5 min (−60 °C to −80 °C) at 5 cm on the surface of the liquid nitrogen, and finally placed directly into the liquid nitrogen for storage. When thawing the frozen sperm, the cryovials were quickly removed from the liquid nitrogen, and put in a 37 °C water bath with shaking and thawing until the ice crystals in the cryovials just melted. Care was taken to avoid local thawing and its impact on sperm quality.

### 2.4. RNA Extraction, cDNA Library Construction, and Sequencing

According to the manufacturer’s protocol, TRIzol reagent (Invitrogen, Waltham, MA, USA) was used to extract total RNA from the *E. lanceolatus* fresh sperm and frozen sperm (El-0, El-23, El-49, and El-61). The quality of RNA was evaluated using the Agilent 2100 bioanalyzer (Agilent Technologies, Santa Clara, CA, USA), and RNase-free agarose gel was used for electrophoretic detection. After the total RNA was extracted, eukaryotic mRNA was enriched with Oligo (dT) beads. The enriched mRNA was fragmented with a buffer and reverse-transcribed into cDNA by using the NEBNext Ultra RNA Library Prep Kit for Illumina (New England Biolabs, Ipswich, MA, USA). The purified double-stranded cDNA fragments were end-repaired, A-base was added, and the fragments were ligated to the Illumina sequencing adapters. The ligation reaction was purified using AMPure XP Beads (1.0×), and the size of the ligated fragment was selected through agarose gel electrophoresis and PCR amplification. RNA samples were sequenced on the Illumina HiSeq^TM^ 6000 platform by Gene Denovo Biotechnology Co. (Guangzhou, China). All reads have been deposited at the National Centre for Biotechnology Information and can be accessed in the SRA database under accession number PRJNA980002.

### 2.5. Data Analyses

Low-quality reads were filtered to obtain clean reads to ensure the reliability of the subsequent analyses. For filtering, the following reads were removed: (1) reads containing adapters; (2) reads containing more than 10% of unknown nucleotides (N); (3) all A-base reads; (4) low-quality reads containing more than 50% of low-quality (Q-value ≤ 20) bases. The clean reads after filtering were mapped to the ribosomal RNA (rRNA) database using Bowtie2 [12]. The rRNA reads were removed. The retained unmapped reads were mapped to the *E. lanceolatus* genome [13] using HISAT2 [14]. Based on the expression results for known mRNA genes in the samples, a principal component analysis was performed and Pearson correlation coefficients were calculated to evaluate repeatability between samples and exclude outlier samples. Sample expression was determined according to the FPKM (reads per kilobase of exon model per million mapped reads). The read count data were obtained from the analysis of mRNA expression levels. DESeq2 [15] was used for the DEG analysis, and the screening conditions were FDR < 0.05 and |log_2_FC| > 2. The DEGs were then subjected to GO and KEGG functional enrichment analyses.

### 2.6. Sample Time-Series Analysis

The STEM (short time-series expression miner) was used to analyze and cluster the DEGs at four time points, providing a visual representation of different gene expression patterns in the fresh and frozen sperm of *E. lanceolatus*. The number of modules was set to 20. Using log_2_ (FPKM), we standardized the data, and *p* < 0.05 was the screening threshold for a reliable trend.

### 2.7. Weighted Gene Co-Expression Network (WGCNA) Construction

Low-quality genes (RPKM value < 1) were filtered out to improve the accuracy of the results. A total of 18,380 genes were imported into the WGCNA to construct co-expression modules. Modules and expression correlation coefficients of the genes were calculated, and GO and KEGG pathway enrichment analyses were further performed. Networks for the visualization of genes associated with the traits of samples, co-expression patterns, and gene interactions were constructed using Cytoscape v3.9.1 [16].

### 2.8. Experimental Validation Using qRT-PCR

To verify the quantitative gene expression levels in the transcriptome sequencing, qRT-PCR was used to analyze 14 genes with high expression in the cryopreserved samples. cDNA was reverse-transcribed from the RNA samples (El-0, El-23, El-49, and El-61) and then used as a template. β-Actin was used as a reference gene [17]. The primers were designed using Primer Premier 5, and the primer sequences are shown in Table 1. The qRT-PCR experiment was conducted using the LightCycler 480II (Roche Diagnostics GmbH, Mannheim, Germany) with TB Green Premix Ex Taq II (Tli RNaseH Plus) (Takara, Kusatsu, Japan) according to the manufacturer’s instructions. The reaction system was 20 μL containing 10 μL of TB Green Premix Ex Taq II (Tli RNaseH Plus) (2×), 0.8 μL of forward primer (10 μM), 0.8 μL of reverse primer (10 μM), 2 μL of template cDNA, and 6.4 μL of ddH_2_O. Reaction conditions were 95 °C for 30 s, 40 cycles (95 °C for 5 s and 60 °C for 30 s), and 50 °C for 30 s. The relative expression patterns were calculated based on the 2^−∆∆Ct^ method and the data are presented as the mean ± SD (*n* = 3).

## 3. Results

### 3.1. Transcriptome Sequencing and Assembly

**Figure 1:**
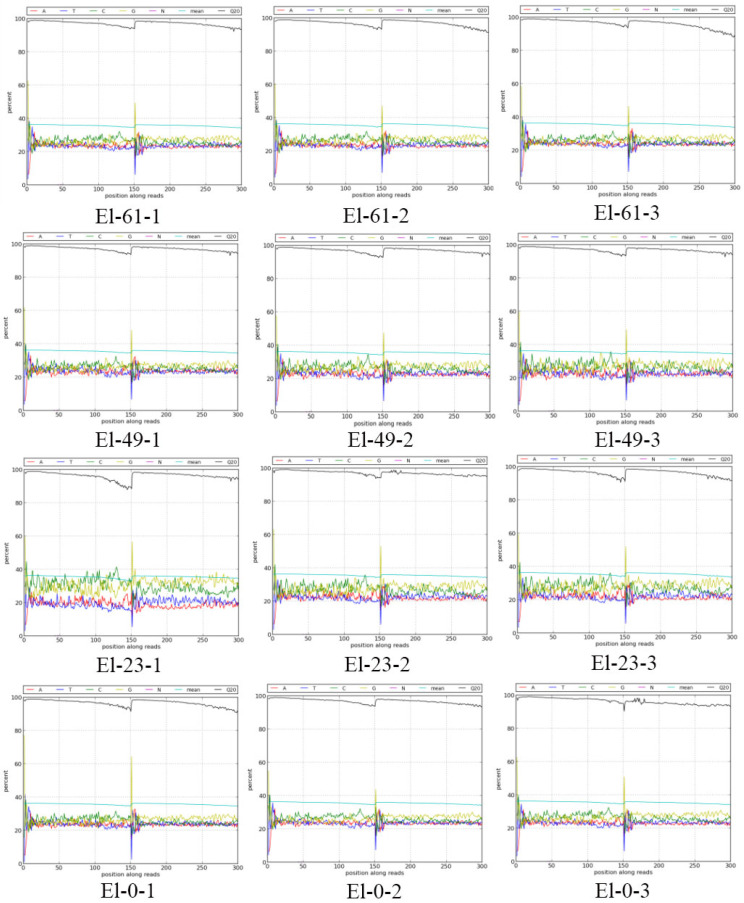
Distribution of bases and mass changes after filtering.

After data filtering and trimming, 9.62 × 10^8^ clean reads were generated from 12 samples in four groups (3 pseudo replicates per group). The Q20 and Q30 values of each sample were greater than 96.70% and 92.08%, respectively (Table 2). All summary statistics suggest that the sequencing quality was high enough for further analyses. The base content and base mass distribution maps were obtained by sequencing the transcriptomes of three sperm samples after cryopreservation (Figure 1). The A-T and C-G bases of the four samples were similar, indicating that the base composition was stable and balanced and the sequencing quality was high. The base masses of the four groups of samples were stable at about 40%, and the proportion of low-quality bases was small, indicating good sequencing quality. The transcriptome results were compared with the whole genome of *E. lanceolatus* as the reference, and the proportion of reads mapped to the genome indicated that the measured species was basically the same as the reference genome (Table 3).

### 3.2. Analysis of Differentially Expressed Genes

**Figure 2:**
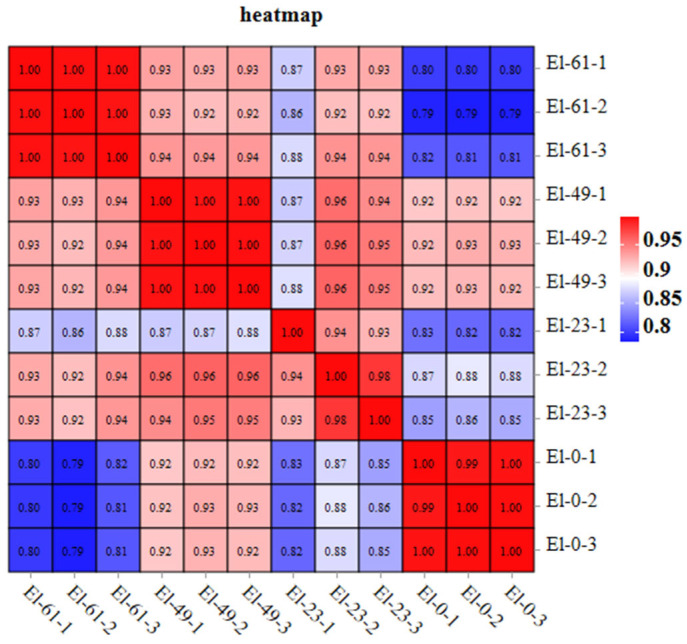
Sample correlation heat map of the mRNA.

Correlation coefficients for the mRNA levels in the 12 samples in this study were above 0.93, indicating a good correlation between the samples (Figure 2).

**Figure 3:**
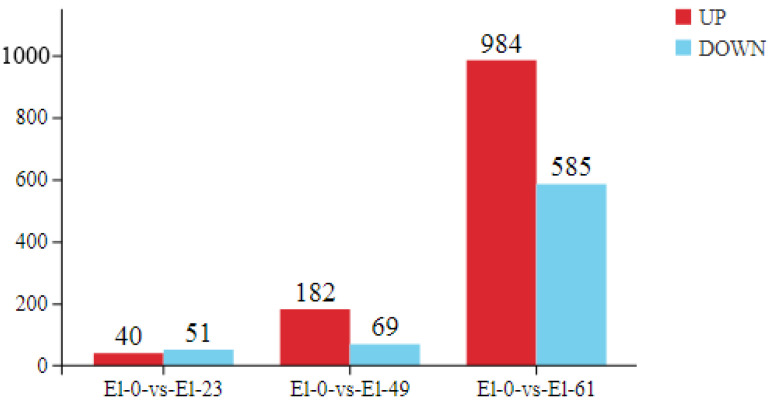

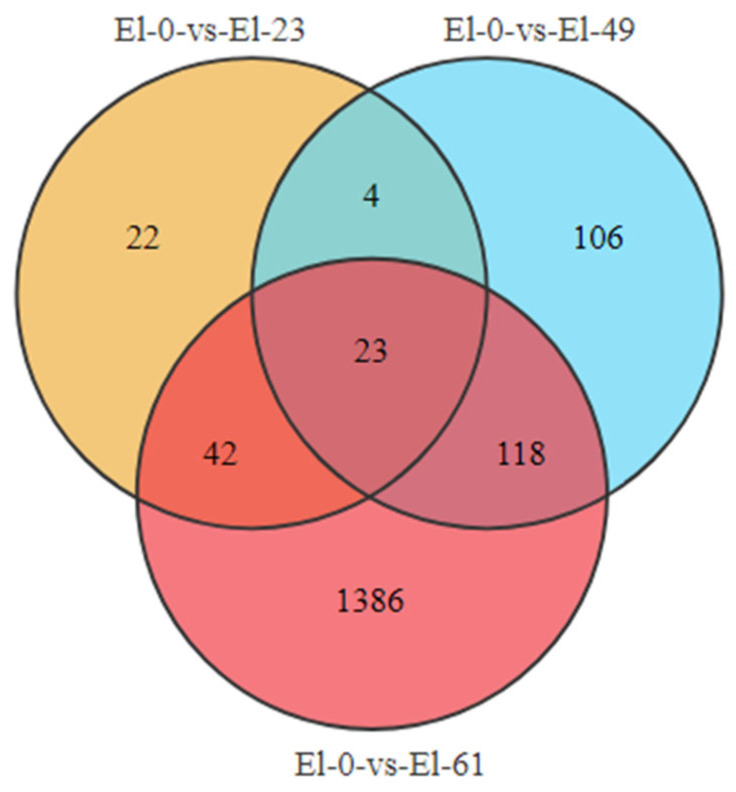
Differential gene plot. The plot on the left shows the number of DEGs identified from the fresh sperm and post-thawed sperm of *E. lanceolatus*. Red indicates upregulated genes and blue indicates downregulated genes. The right shows a Venn diagram depicting the distribution of DEGs between the fresh and post-thawed sperm of *E. lanceolatus*.

We used an FDR < 0.05 and a fold change > 2 as the criteria to screen for DEGs in pairwise comparisons between the fresh sperm and frozen sperm. A total of 1911 differentially expressed mRNAs were identified, including 91 in El-0-vs-El-23 (40 upregulated and 51 downregulated), 251 in El-0-vs-El-49 (182 upregulated and 69 downregulated), and 1569 in El-0-vs-El-61 (984 upregulated and 585 downregulated). Among these, 23 common DEGs were found, which were mainly enriched in the MAPK signaling pathway, responsible for regulating physiological processes such as cell growth, differentiation, apoptosis, and death (Figure 3).

### 3.3. GO and KEGG Analyses of DEGs

According to a GO function annotation analysis, the DEGs were classified into 39 GO terms in El-0-vs-El-23, including 19 biological processes, 9 molecular functions, and 11 cellular components. The fewest DEGs were obtained in El-0-vs-El-23, and these were mainly enriched in biological processes, particularly cellular processes, followed by binding, single-organism processes, and biological regulation. A total of 19 significant terms were screened, such as biological regulation, pilus assembly, animal organ development, nucleic acid binding transcription factor activity, and phosphoric ester hydrolase activity.

According to the GO functional annotation analysis, the DEGs were classified into 44 GO terms in El-0-vs-El-49, including 20 biological processes, 9 molecular functions, and 15 cellular components. These DEGs were mainly enriched in biological processes, particularly cellular processes. The DEGs were enriched for binding in the molecular function category, followed by cellular processes. A total of 20 significant terms were screened, such as cyclin-dependent protein serine/threonine kinase inhibitor activity, enzyme inhibitor activity, and cell cycle arrest.

**Figure 4:**
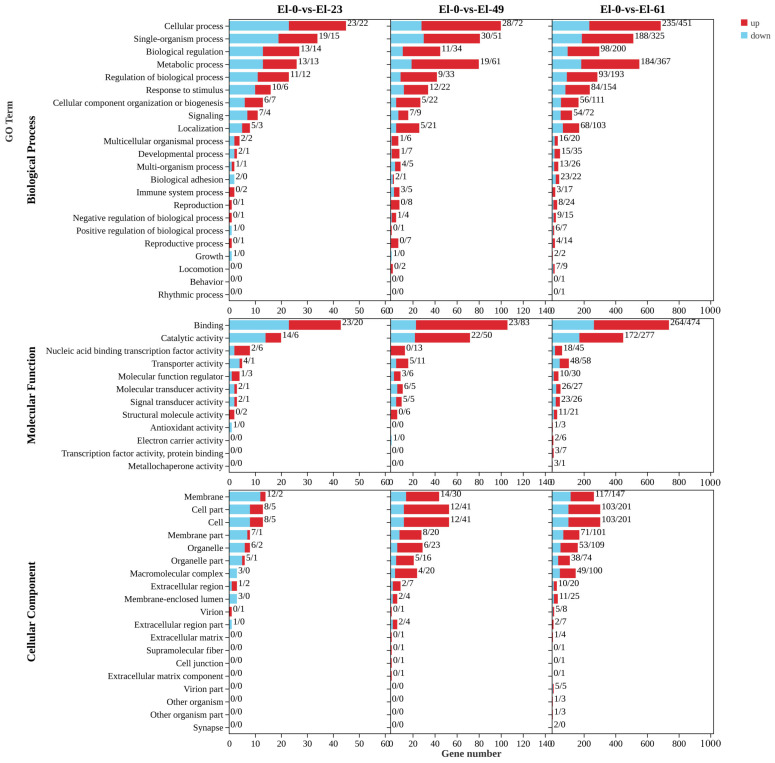
GO term enrichment in fresh sperm (El-0) and three groups of post-thawed sperm (El-23, El-49, and El-61) of *E. lanceolatus*.

According to the GO functional annotation analysis, the DEGs were classified into 53 GO terms in El-0-vs-El-61, including 22 biological processes, 12 molecular functions, and 19 cellular components. The most DEGs were obtained in El-0-vs-El-23. These genes were mainly enriched for binding in the molecular function category. A total of 20 significant terms were screened, such as protein kinase inhibitor activity, kinase inhibitor activity, and the regulation of metabolic processes. In all comparisons, the DEGs showed the least enrichment for the cell fraction (Figure 4).

KEGG is the main public database with pathway information and can provide additional insights into the biological functions of DEGs. Therefore, all DEGs in the giant grouper sperm were evaluated using the KEGG pathway database to predict biological functions. A total of 132 KEGG pathway terms were enriched in El-0-vs-El-23, including 19 significant terms (*p* < 0.05). These included the JAK-STAT signaling pathway, apoptosis, the MAPK signaling pathway, and sulfur metabolism.

A total of 216 KEGG pathways were enriched in El-0-vs-El-49, including 43 significant terms (*p* < 0.05). These included the IL-17 signaling pathway, apoptosis, the MAPK signaling pathway, and the P53 signaling pathway.

**Figure 5:**
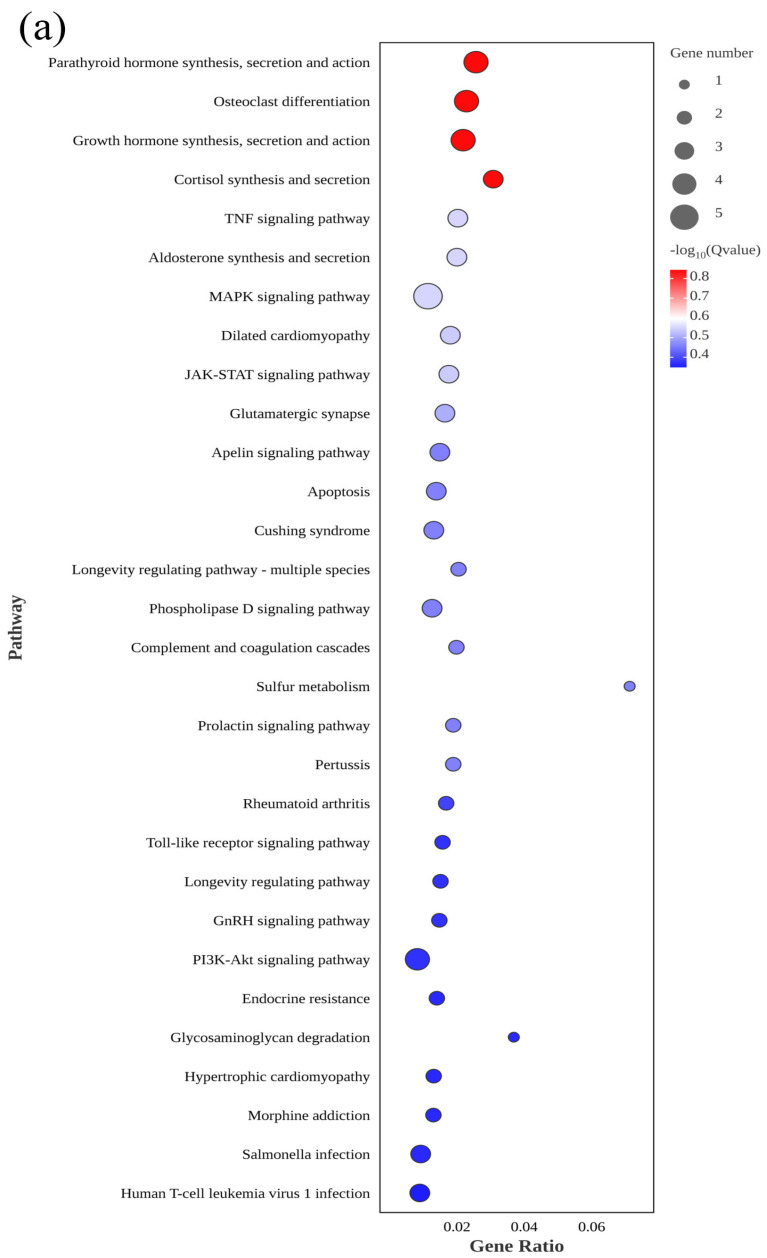

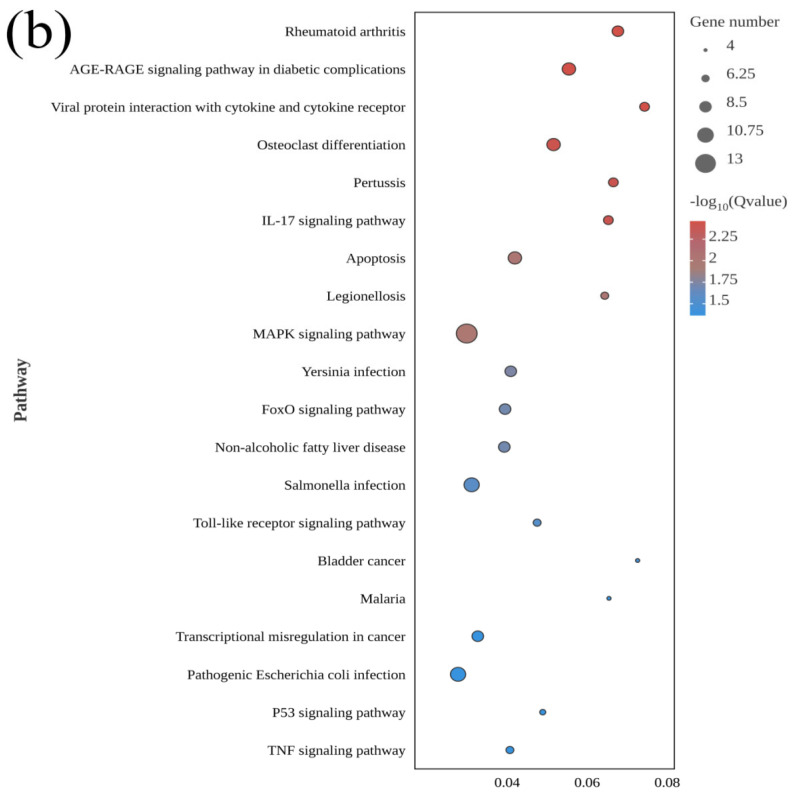

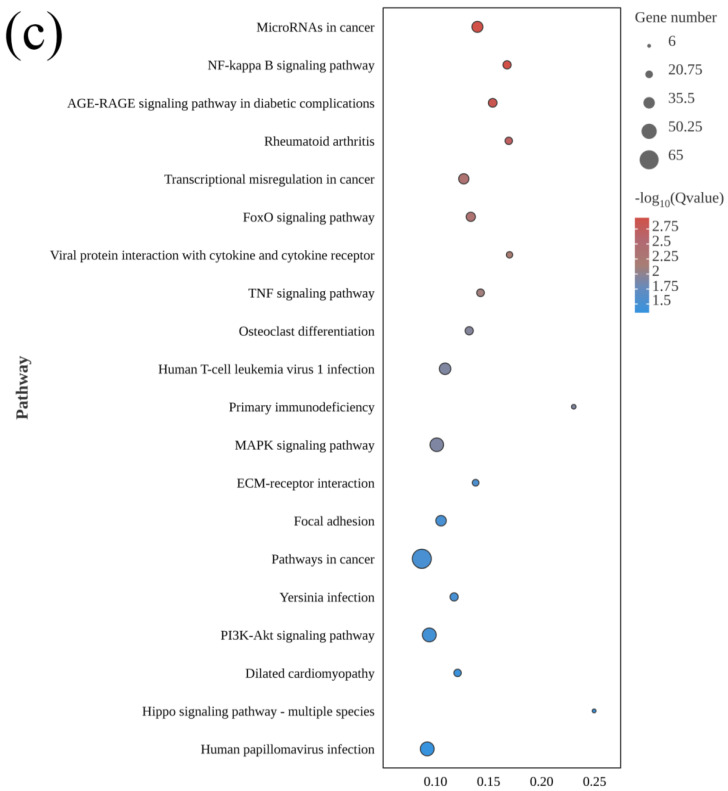
KEGG pathway enrichment analysis of differentially expressed genes between fresh sperm (El-0) and three groups of post-thawed sperm (El-23, El-49, El-61) of *E. lanceolatus*. (**a**) KEGG pathway enrichment analysis of El-0-vs-El-23. (**b**) KEGG pathway enrichment analysis of El-0-vs-El-49. (**c**) KEGG pathway enrichment analysis of El-0-vs-El-61.

A total of 312 KEGG pathways were enriched in El-0-vs-El-61, including 72 significant terms (*p* < 0.05). These included the following: osteoclast differentiation, the PI3K-AKt signaling pathway, the MAPK signaling pathway, and the Hippo signaling pathway. The MAPK signaling pathway was enriched in all three comparisons (Figure 5).

### 3.4. Sample Time-Series Analysis of DEGs

**Figure 6:**
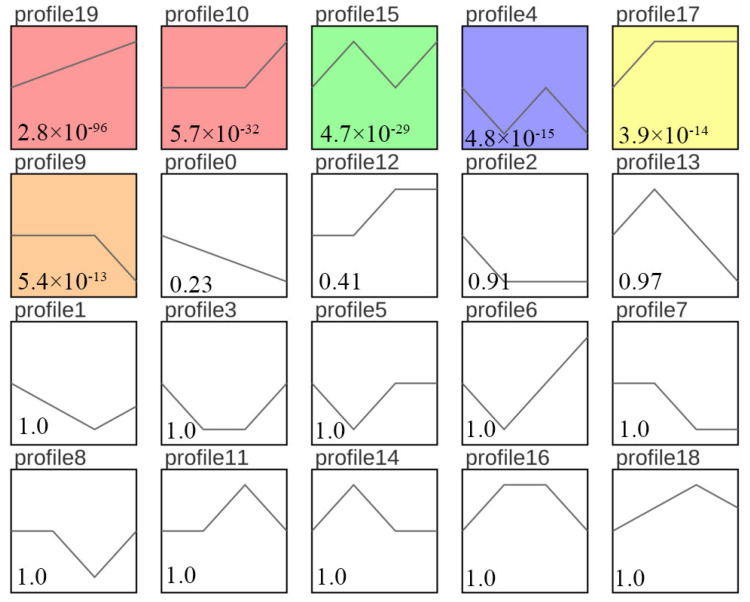
Expression trend analysis for DEGs between fresh sperm (El-0) and three groups (El-23, El-49, El-61) of post-thawed sperm of *E. lanceolatus*. The colors indicate significant enrichment; white indicates no significant enrichment. The number above each box represents different trends. The *p*-value is shown in the lower-left corner.

To comprehensively evaluate the effects of different freezing times on sperm, we analyzed the expression trends of DEGs and selected biologically meaningful target genes with *p* < 0.05 as the screening condition. (Note that gene expression was expressed as FPKM, and log_2_ normalization was performed according to the expression level in the first sample.) The DEGs were assigned to 20 modules, of which 6 modules were significant (*p* < 0.05). The overall gene expression trend was classified as either rising or falling. The DEGs in profile 19 were consistently upregulated (Figure 6). These findings effectively revealed the gene expression status of giant grouper sperm after long-term cryopreservation.

**Figure 7:**
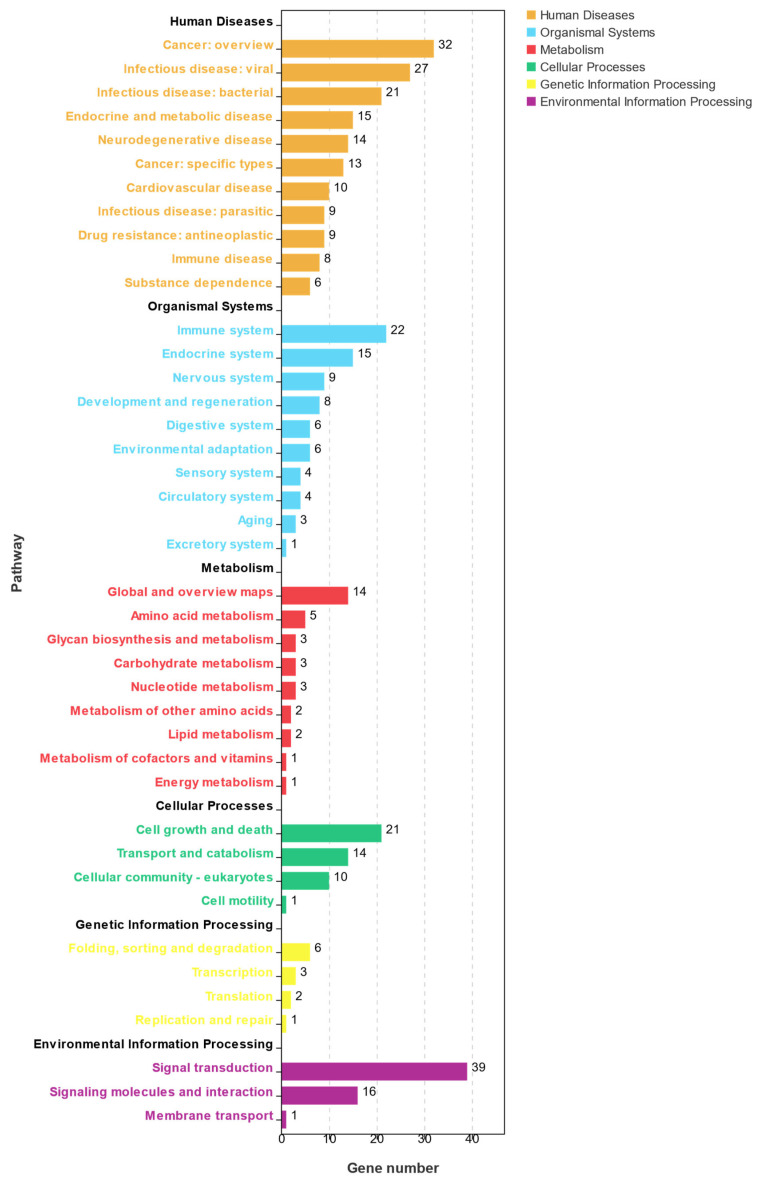
KEGG pathway analysis of DEGs showing continuous upregulation in the post-thawed sperm of *E. lanceolatus*.

A KEGG enrichment analysis of the DEGs in profile 19 is summarized in Figure 7. A total of 231 KEGG pathways were enriched in profile 19, and 48 KEGG pathways were significantly enriched (*p* < 0.05). These pathways were divided into six classes: human disease, metabolism, organism system, genetic information processing, environmental information processing, and cellular processes. Most of the pathways were related to cell growth and death, the immune system, the endocrine system, and signal transduction.

### 3.5. Construction of the Co-Expression Network (the WGCNA)

**Figure 8:**
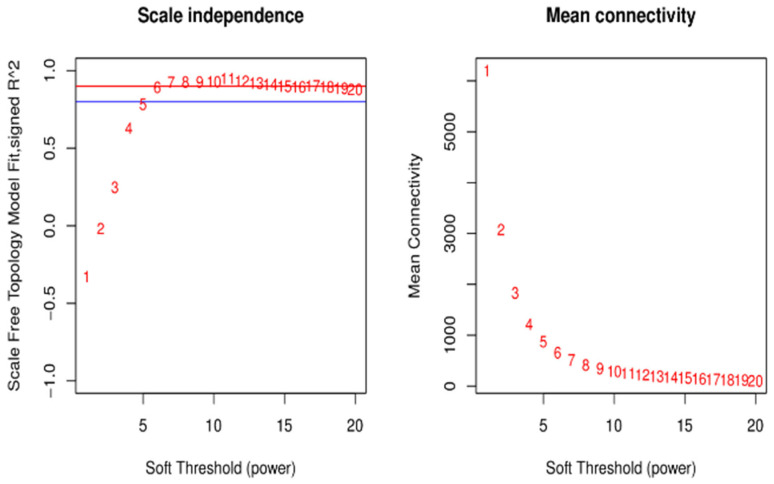
Soft threshold determination for the gene co-expression network. Notes: The left panel shows the scale-free network fitting index under different soft thresholds; the right panel shows the network connectivity under different soft thresholds. The red line indicates the correlation coefficient is 0.9.

Figure 8 left shows the selected soft threshold of 6, and Figure 8 right shows the network connectivity for different soft thresholds for the construction of co-expression networks (Figure 8).

#### 3.5.1. Clustering and Module Cutting of the Co-Expression Networks

**Figure 9:**
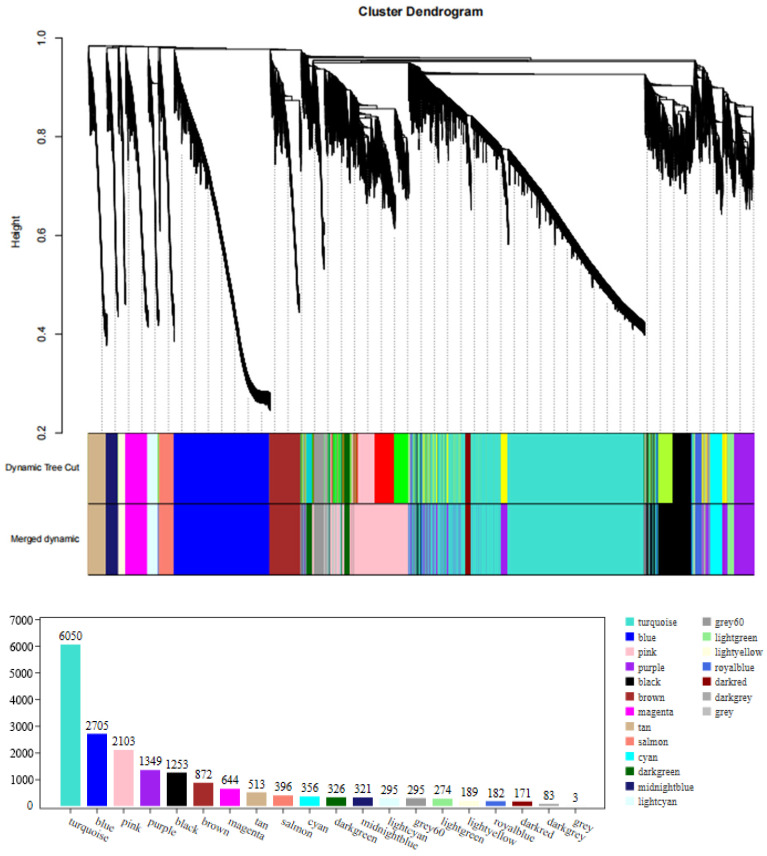
Clustering dendrograms of 18,380 genes. Dissimilarity was based on topological overlap, together with the assigned module colors. The 20 co-expression modules are shown in different colors.

**Figure 10:**
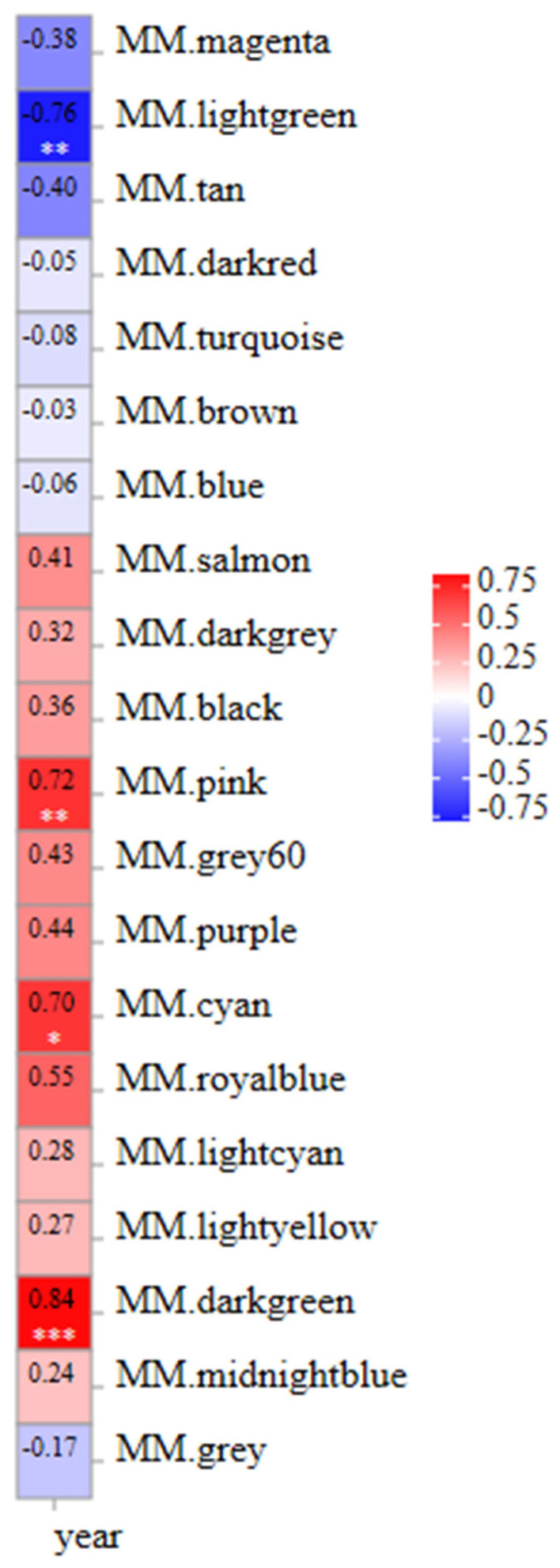
Correlation heat map of module properties associated with the freezing time. “*” is means *p* < 0.05, “**” is means *p* < 0.01, “***” is means *p* < 0.001.

A weighted gene co-expression network of DEGs between fresh and frozen *E. lanceolatus* sperm was generated. The genes with RPKM values of <1 were removed, with a total of 18,380 genes remaining in the network after screening, divided into 20 modules (Figure 9). The turquoise module had the largest number of genes (i.e., 6050), and the smallest gray module had 3 genes (Figure 9). Finally, in a correlation analysis between all modules and the freezing length, the dark-green module had a high positive correlation, followed by the pink module and cyan module, and the light-green module had a negative correlation (Figure 10).

#### 3.5.2. GO and KEGG Enrichment Analyses of Genes in the Dark-Green Module

**Figure 11:**
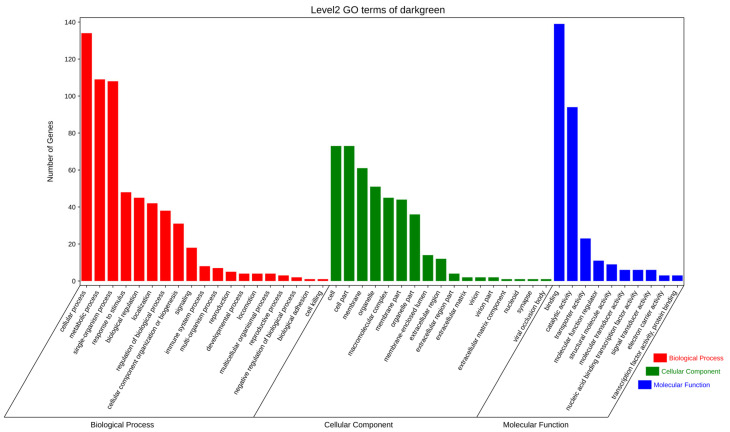
GO term enrichment analysis of DEGs between fresh sperm (El-0) and three groups (El-23, El-49, El-61) of post-thawed sperm of *E. lanceolatus* in the dark-green module.

To further explore the potential functions of the significantly enriched modules, GO and KEGG enrichment analyses were used to evaluate genes in the dark-green module. The GO functional enrichment analysis revealed terms in the following three main categories: biological processes, molecular functions, and cellular components. In particular, genes were enriched in cellular processes, metabolic processes, binding, and individual biological processes (Figure 11).

**Figure 12:**
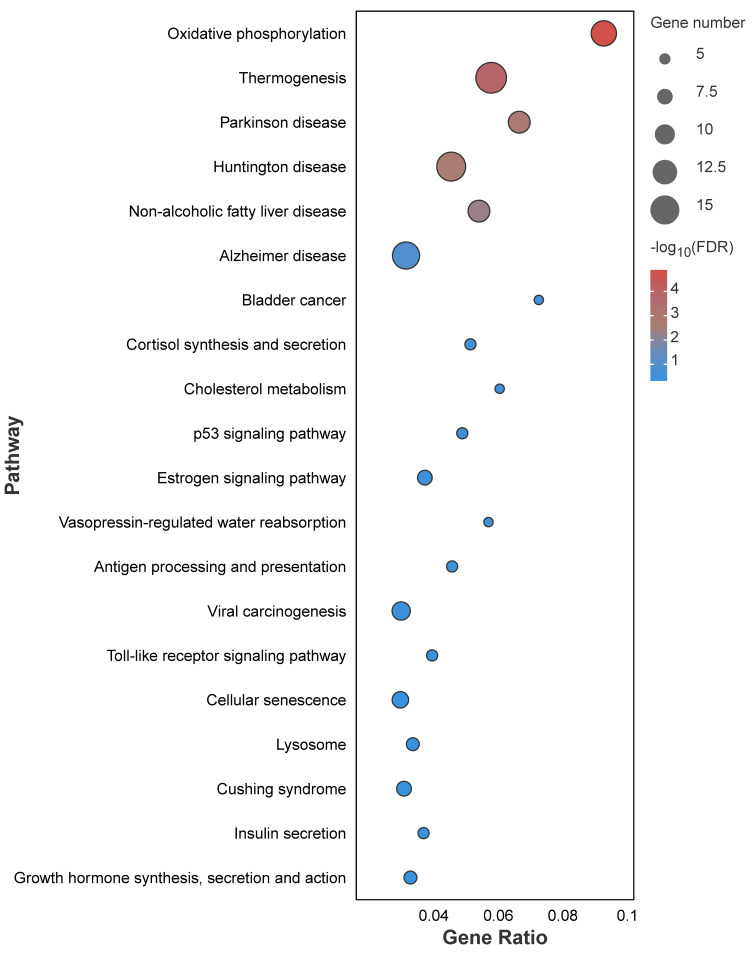
KEGG pathway enrichment analysis of DEGs between fresh sperm and three groups of post-thawed sperm of *E. lanceolatus* in the dark-green module.

The KEGG pathway enrichment analysis of the dark-green module showed that a total of 231 pathways were detected, of which 23 were significantly enriched (*p* < 0.05); these included the P53 signaling pathway, oxidative phosphorylation, cellular senescence, and other pathways (Figure 12).

#### 3.5.3. Gene Co-Expressed Network of the Dark-Green Module

**Figure 13:**
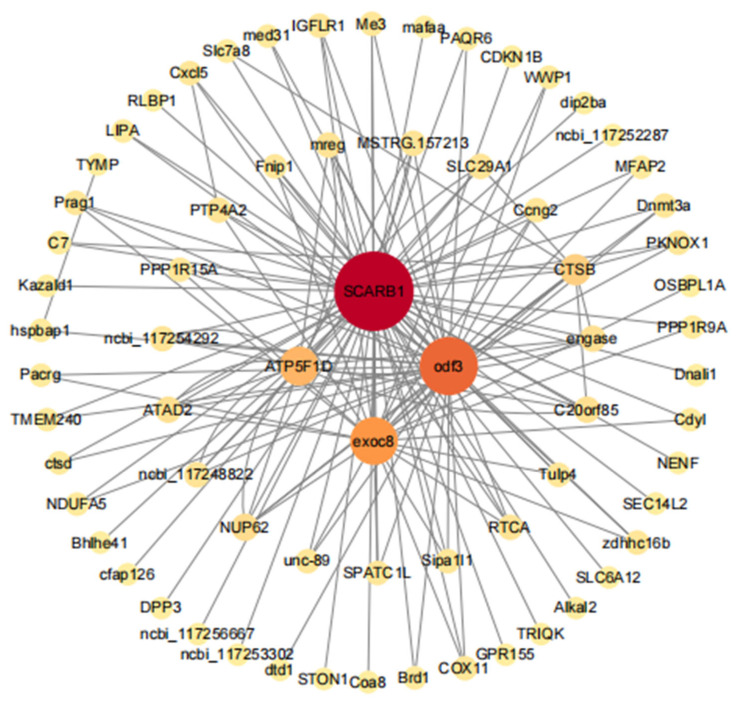
Network relationships for genes in the fresh sperm and three groups of post-thawed sperm of *E. lanceolatus* in the dark-green module.

We used genes with the top 10 module membership (MM) values in the dark-green module for a network analysis using Cytoscape. The top 150 pairs were selected for the construction of a co-expression network. The genes with the highest connectivity in the network were the core genes. The nodes with the top four degree values were selected as the core genes of the co-expression network of this module. Four genes, *scarb1*, *odf3*, *exoc8*, and *atp5f1d*, were found at the core of the network. These genes were mainly involved in immune regulation, sperm movement, and mitochondrial energy conversion (Figure 13).

### 3.6. Validation of Gene Expression via qRT-PCR

**Figure 14:**
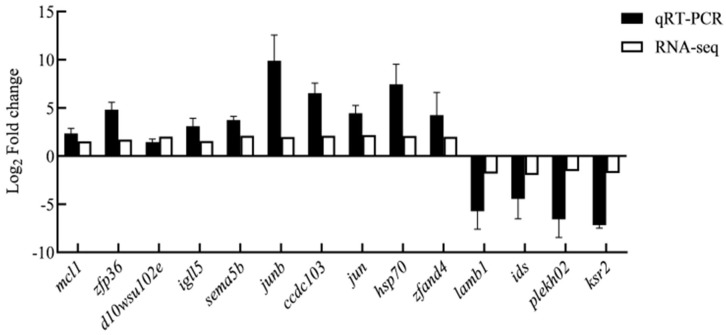
Relative expression levels of differentially expressed genes determined using real-time quantitative PCR and RNA-seq.

To reveal the key genes associated with fresh sperm and post-thawed sperm, 14 highly expressed genes under cryopreservation were selected for verification with RT-qPCR. The results were similar to those obtained through sequencing, confirming the reliability of the sequencing data (Figure 14).

## 4. Discussion

In the study of cryodamage to the giant grouper sperm by cryopreservation, we have previously found that ultra-low-temperature cryopreservation affects sperm motility, plasma membrane integrity, enzyme activity, and ultrastructural properties, and these problems increase gradually with the freezing time. These findings suggest that sperm quality decreases as the storage duration increases. This is likely caused by unstable cryopreservation conditions. Sperm inactivation is mainly due to the production of large amounts of ROS, DNA damage, plasma membrane damage, and mitochondrial damage [18,19,20]. This is also in agreement with our previous experimental results [7]. There are few reports of RNA damage in sperm during frozen storage. Conventional views suggest that sperm are highly differentiated cells with less cytoplasm and condensed nuclei, and transcription and translation are inactive [21,22]. Various mRNAs in sperm associated with biological processes, such as sperm motility, fertilization, and early embryonic development, have been identified [23]. To elucidate the specific variation in sperm freezing damage in *E. lanceolatus*, we used a transcriptome analysis to explore the gene regulatory network. In this study, we used sperm samples taken from the same fish at different times; mainly considering the fact that these same-fish sperm samples had a consistent genetic background, the transcriptome analysis results for samples with different freezing times were comparable. The giant grouper has a long life span, and our giant grouper was in its youth when we took the semen samples. At the same time, the sperm vitality was checked and a sperm vitality of more than 85% was selected for sperm cryopreservation. Therefore, the fish itself aging would not have had a significant effect on the effect of sperm cryopreservation. Of note, 12 cDNA libraries at four different freezing time points were established for transcriptome sequencing.

### 4.1. Differential Expression between Fresh and Frozen Sperm of E. lanceolatus

Cryopreserved sperm is used for artificial reproduction, seed production, and breeding. In this study, 1911 DEGs were identified, with an increase from 91 to 1569 as the cryopreservation time increased for the giant grouper sperm. With the extension of the freezing time, the number of DEGs increased gradually. DEGs in the frozen sperm increased significantly after freezing for 61 months. These results provide valuable information and a basis for further studies on fish sperm cryoinjury.

GO enrichment analyses of the DEGs between the fresh and frozen sperm of *E. lanceolatus* revealed enrichment for phospholipid hydrolases, enzyme inhibitor activity, cell cycle arrest, and the regulation of metabolic processes. It is speculated that cryogenic freezing is related to sperm enzyme activity, cell metabolism, and death. A Yunnan semi-fine wool sheep sperm transcriptome analysis also showed enrichment for similar biological processes [24]. Sperm will produce a large amount of ROS after cryopreservation. Several studies have reported an increase in ROS production after cryopreservation in different species, impairing cell viability and motility [6,25]. Elevated oxidative stress is often associated with cell death [26]. Therefore, sperm can regulate ROS production via metabolic processes.

Our KEGG pathway analysis of the fresh and frozen sperm of *E. lanceolatus* revealed that DEGs were involved in the MAPK signaling pathway, the P53 signaling pathway, the JAK-STAT signaling pathway, and apoptosis. With the prolonged cryopreservation time, significantly enriched signaling pathways increased gradually. Of note, the DEGs in the three pairwise comparisons of giant grouper fresh sperm and frozen sperm were involved in the MAPK signaling pathway. This suggests that the MAPK signaling pathway plays an important role in the whole process of the long-term freezing of sperm. Mitogen-activated protein kinase (MAPK) is a crucial part of the cellular signal transduction pathway and can be activated by environmental stimuli to contribute to the regulation of cell proliferation, differentiation, growth, and apoptosis [27]. MAPK, together with MAPKK and MAPKKK, constitute the MAPK signaling pathway [28]. The MAPK signaling pathway has important effects on cell growth, stress, and the immune response [29]. The MAPK signaling pathway contributes to the response to low temperatures in both animals and plants, such as *Larimichthys crocea* [30], *Solanum tuberosum* L. [31], and the pearl gentian grouper (*E. lanceolatus*♂ × *E. fuscoguttatus*♀) [32]. In humans, sperm motility is regulated via the MAPK signaling pathway [33]. In addition, more DEGs involved in the regulation of the MAPK signaling pathway were detected with the extension of the freezing time, such as *nr4a1*, *hsp70*, and *traf2*.

*hsp70* is important for protein repair and degradation [34], participates in various metabolic processes in cells [35], enhances antioxidant activity [36], and protects against stress [37]. In this study, *hsp70* gene expression in the fresh sperm differed significantly from that in the frozen sperm. Expression levels increased initially and then decreased. It is speculated that the sperm exhibited stress resistance during cryopreservation, with the stress resistance decreasing over the 61 months. Polymorphism in the *hsp70* gene is significantly correlated with cold tolerance in the GIFT tilapia (*Oreochromis niloticus*) [38]. The *nr4a1* gene is a member of the nucleus receptor family that can regulate various physiological processes, including cell growth, metabolism, and immunity, and participates in the cell cycle [39]. In this study, *nr4a1* gene expression differed significantly between the fresh sperm and frozen sperm at 61 months. The *nr4a1* gene may be involved in sperm metabolism during cryopreservation. The *traf2* gene is involved in the regulation of cell apoptosis. It is a member of the tumor necrosis factor (TNF) family and can lead to the activation of the MAPK signaling pathway, thus regulating the biological activities of cells [40]. In this study, *traf2* gene expression decreased after sperm cryopreservation. This gene is clearly involved in the regulation of immune defenses in sperm after cryopreservation. All three genes have established roles in stress resistance in different species [41,42,43].

### 4.2. Time-Series Expression Profiles and Co-Expression Network of Differentially Expressed Genes between Fresh Sperm and Frozen Sperm of E. lanceolatus

A comparative analysis of different time points combined with a time-series analysis revealed genes with expression changes over time during sperm cryopreservation, such as *bcl2*. The Bcl-2 family genes can lead to irreversible apoptosis [44]. In this study, *bcl2* gene expression differed significantly between the fresh sperm and frozen sperm at 61 months. With the extension of the freezing time, the freezing damage increased gradually, and sperm damage reached its maximum at 61 months. Time-series expression profiling revealed that DEGs that were continuously upregulated were closely related to cell growth and death, amino acid metabolism, the immune system, the endocrine system, and signaling. DEGs were involved in the IL-17 signaling pathway, the TNF signaling pathway, the T-cell receptor signaling pathway, and apoptosis. Cell death is usually divided into three types: apoptosis, necrosis, and autophagy [45]. Apoptosis is a type of programmed cell death that can maintain the stability of the intracellular environment and improve adaptation to environmental conditions. Mitochondrial apoptosis is one of the most common forms of cell apoptosis and can produce large amounts of energy [46]. Through transmission electron microscopy of the giant grouper sperm, substantial mitochondrial damage was observed; it is speculated that the mitochondria play a crucial role in the progression of apoptosis. Low-temperature stress causes apoptosis, as observed in *Rachycentron canadum* [47], *Ictalurus furcatus* [48], and *Marsupenaeus japonicus* [49]. These findings indicate that during the cryopreservation of sperm, low temperatures will stimulate the sperm to activate immune-related genes for protection, and low temperatures will also cause sperm damage.

Because most genes interact with various other genes, the combination of RNA-seq and the WGCNA has become a critical, cost-effective approach to discover hub genes and interactions that might be functionally related to stress [26]. In this study, the WGCNA revealed that the dark-green module was the most significant in terms of the sperm response to cryopreservation. Our KEGG pathway analysis of genes in the dark-green module indicated that these genes were involved in the P53 signaling pathway, oxidative phosphorylation, and cellular senescence. Four hub genes were found in the network: *scarb1*, *odf 3*, *exoc8*, and *atp5f1d*.

*Scarb1* is a key membrane transporter gene encoding a steroid synthesis precursor and can transport cholesterol efficiently. It is a key regulatory gene participating in many physiological activities and immune regulation; as a member of the CD36 family, it has been widely studied in human diseases [50]. Cholesterol is an important structural component in the maintenance of cell membrane permeability and fluidity and is a precursor for steroid synthesis, which is essential for normal sperm production [51]. In rat cardiomyocytes subjected to hypoxia/reoxygenation, we found that *scarb1* could be upregulated through the inhibition of miR-125a-5p, thus exerting a protective effect [52]. In this study, the *scarb1* gene had the highest connectivity in the module. Combined with the physiological role of this gene, we speculate that it plays a key role in steroid metabolism and membrane transport during sperm freezing.

The integrity of the sperm flagellar structure is a prerequisite for sperm motility. The odf (outer dense fiber) structure is one of the “9 + 2” microtubule structures produced by sperm flagella. The *odf3* gene encodes one of the major proteins in the odf family; these proteins make up dense peripheral fibers [53]. The odf protein has some effects on the formation of the sperm tail structure and sperm motility, and a decrease in its expression may explain the decrease in sperm motility [54]. Previous research has shown the giant grouper sperm flagella also shows the “9 + 2” structure, and cryopreservation may influence the giant grouper sperm flagella.

Exoc8 can transport intracellular vesicles to the plasma membrane for fusion and plays important roles in several important cellular processes, such as ciliogenesis, cell autophagy, and cytokinesis [55]. Interestingly, some studies have found that exoc8 is closely associated with neural development [56]. A transcriptome analysis of cryopreserved kelp grouper (*Epinephelus moara*) embryos showed that the expression of some genes encoding proteins that protect neurons from the toxic effects of high concentrations of extracellular neurotransmitters decreased significantly, suggesting that cryopreservation influences the central nervous system of larvae [57]. Therefore, the process of long-term cryopreservation may have had the same effect on the giant grouper sperm. In addition, *exoc8* mutations may be associated with cilia, which can assemble on different cell types, including sperm [58]. Based on the effect of *odf3* on sperm flagella, cryopreservation likely has a substantial effect on sperm flagella.

ATP synthase is a key enzyme in the mitochondrial energy conversion process, and its expression level is directly related to the energy metabolism of the mitochondria. *atp5f1d* is a member of the ATP synthases, responsible for ATP production and transport; it plays a key role not only in spermatogenesis but also in sperm capacitation and sperm Ca^2+^ signaling [59]. It is also related to mitochondrial respiration and the maintenance of the normal structure and function of the heart [60]. Decreases in ATP synthase protein expression also affect mitochondrial energy metabolism and fertilization [61]. In previous studies, we found that the cryopreserved sperm ATP content decreases significantly. Therefore, cryopreservation may cause damage to the sperm mitochondria, leading to a decrease in ATP synthase expression and a decrease in the ATP content in the sperm, thus reducing sperm motility in the giant grouper. In conclusion, functional studies of the four hub genes revealed that cryopreservation can have certain effects on sperm mitochondria and flagella, contributing to our understanding of the molecular mechanisms underlying sperm cryoinjury in the giant grouper species.

## 5. Conclusions

We investigated, for the first time, gene expression changes in giant grouper sperm during long-time cryopreservation using RNA-seq. A differential expression analysis revealed 1911 DEGs, including 1206 upregulated genes and 705 downregulated genes. GO and KEGG pathway enrichment analyses revealed that the DEGs were mainly related to pilus assembly and enzyme inhibitor activity and were involved in the MAPK pathway, the P53 signaling pathway, and apoptosis. All of the DEGs were related to sperm freezing injury. In a trend analysis, the gene modules showing consistent upregulation were also significantly enriched in pathways related to apoptosis and immunity. Finally, using the WGCNA, the dark-green module was found to be the most significantly associated with freezing damage. By mapping the gene network within this module, we found that *scarb1*, *odf3*, *exoc8*, and *atp5f1d* may play important roles in the response to freezing. These results provide rich insights into the genetic changes during cryopreservation and the mechanisms underlying cryodamage.

## Figures and Tables

**Table 1:** qRT-PCR primer sequences.

Symbol	Forward Primer	Reverse Primer
*ids*	GGATGGTAAACTCCACGCCA	ACATCATTGTTGGCCCGACT
*junb*	TTCTACAACCGGGGCATCAC	GTAACTCCACCGGCTCCAAG
*plekho2*	ACTCGATCAAGGCCCAAAGG	TCCAAGCGCAGGATACCATC
*hsp70*	TCAATGACTCCCAGCGACAG	TTGTCCAGGCCGTATGCAAT
*Ccdc103*	CGAGGTTGGAGCGTGATGTC	TTTCGCTTCGTTCTCTCGCT
*ksr2*	TCCCTCAAAGATCACCAAGGA	CGTCTGACTGATGTGCAGGT
*sema5b*	TCGTGCTGATCATCTGTTCGT	TGGGAGAACTCCGACATCCA
*jun*	AGAAAGCGGATGAGGAACCG	GACGCGAGCTCCGAATTTTG
*zfp36*	GCAGTAAGTGCCAGTTTGCC	CGTAGGGGCAGTAGCCAAAG
*d10wsu102e*	AGCCAAAGACTGGCAGATCC	GCTGCTGAAGCTTCTCGTTG
*mcl1*	CGAAGGACTCTCACAACGGG	CGGAGGTTCTTGGTCGCATA
*lamb1*	AACCCCAAGCACTCTTACGG	GTCGCATGCGTGACATTTGA
*Zfand4*	TCGCCTCTTTCGTTCACTCC	GAAGGTGGGGAGGGGTCTAT
*igll5*	GCAGTGGGATCTCTACCAGC	CTGGGAGCCCAAAGACACTT
*β-actin*	CTCTGGGCAACGGAACCTCT	GTGCGTGACATCAAGGAGAAGC

**Table 2:** Summary statistics for the raw reads before and after filtering.

Sample	Raw Data (bp)	Clean Data (bp)	Q20 (%)	Q30 (%)	GC (%)
El-61-1	11,503,797,600	11,260,881,272	97.19%	93.07%	52.88%
El-61-2	12,572,943,600	12,403,629,774	97.01%	92.46%	53.00%
El-61-3	11,964,763,200	11,764,204,113	97.16%	92.77%	52.10%
El-49-1	11,607,607,500	11,310,292,815	97.22%	92.95%	52.59%
El-49-2	12,134,965,800	11,901,108,789	97.20%	92.91%	53.89%
El-49-3	11,793,498,600	11,588,109,505	97.12%	92.74%	54.43%
El-23-1	11,899,454,100	11,501,842,347	96.70%	92.33%	61.06%
El-23-2	11,563,097,700	11,268,692,649	96.94%	92.31%	55.27%
El-23-3	12,992,697,600	12,752,937,675	96.86%	92.08%	55.99%
El-0-1	12,138,655,500	11,919,323,093	97.47%	93.30%	52.50%
El-0-2	12,979,071,300	12,533,244,899	96.99%	92.49%	52.80%
El-0-3	11,868,128,400	11,614,133,308	96.89%	92.13%	53.61%

Note: El-0-1, El-0-2, El-0-3 are the fresh sperm, El-23-1, El-23-2, El-23-3 are the frozen sperm from the 23rd month, El-49-1, El-49-2, El-49-3 are the frozen sperm from the 49th month, and El-61-1, El-61-2, El-61-3 are the frozen sperm from the 61st month.

**Table 3:** Comparison of reference statistics for the reference genomes.

Sample	Total	Unique_Mapped (%)	Multiple_Mapped (%)	Total_Mapped (%)
El-61-1	60,355,436	36,288,656 (60.12%)	5,790,104 (9.59%)	42,078,760 (69.72%)
El-61-2	65,623,376	38,386,462 (58.50%)	6,137,785 (9.35%)	44,524,247 (67.85%)
El-61-3	63,992,594	38,520,927 (60.20%)	6,298,505 (9.84%)	44,819,432 (70.04%)
El-49-1	51,620,698	29,550,592 (57.25%)	3,952,694 (7.66%)	33,503,286 (64.90%)
El-49-2	52,834,236	29,249,147 (55.36%)	4,232,201 (8.01%)	33,481,348 (63.37%)
El-49-3	48,739,846	27,147,933 (55.70%)	3,759,760 (7.71%)	30,907,693 (63.41%)
El-23-1	47,965,958	20,982,137 (43.74%)	3,701,637 (7.72%)	24,683,774 (51.46%)
El-23-2	52,571,466	26,721,876 (50.83%)	4,236,098 (8.06%)	30,957,974 (58.89%)
El-23-3	59,635,270	31,264,050 (52.43%)	4,801,141 (8.05%)	36,065,191 (60.48%)
El-0-1	63,585,244	35,390,829 (55.66%)	4,999,935 (7.86%)	40,390,764 (63.52%)
El-0-2	63,890,574	35,368,631 (55.36%)	4,969,086 (7.78%)	40,337,717 (63.14%)
El-0-3	59,705,142	32,478,564 (54.40%)	4,660,800 (7.81%)	37,139,364 (62.20%)

## Data Availability

All reads have been deposited at the National Centre for Biotechnology Information and can be accessed in the SRA database under accession number PRJNA980002.
